# Quadriceps tendon autograft with patellar bone block and semitendinosus augmentation demonstrates predictable midterm outcomes for patellar tendon disruption following primary total knee arthroplasty

**DOI:** 10.1002/jeo2.70730

**Published:** 2026-05-04

**Authors:** Ashok Rajgopal, Saksham Tripathi, Manish Kumar Singh, Kalpana Aggarwal

**Affiliations:** ^1^ Institute of Musculoskeletal Disorders and Orthopaedics, Medanta—The Medicity Hospital Gurugram Haryana India; ^2^ Institute of Education and Research, Medanta—The Medicity Hospital Gurugram Haryana India

**Keywords:** extensor mechanism allograft, patellar tendon disruption, quadriceps tendon with patellar bone block autograft, semitendinosus augmentation, total knee arthroplasty

## Abstract

**Purpose:**

Patellar tendon (PT) disruption following primary total knee arthroplasty (TKA) is an uncommon but devastating complication, with current available surgical options reporting fair to poor long‐term results. We report our experience using the quadriceps tendon autograft with patellar bone block and semitendinosus augmentation (QTPBSTA) as an alternate graft option, at a mean follow‐up of 6.9 ± 3.6 years.

**Methods:**

This retrospective study cohort included 29 patients with isolated PT ruptures following primary TKA with unresurfaced patellae, between January 2010 and December 2023, treated using the QTPBSTA technique. The diagnosis was confirmed using ultrasonography in 21 patients and magnetic resonance imaging in 8 patients. All the patients were retrospectively evaluated for outcomes at a midterm follow‐up. Data collection included pre‐ and post‐operative extensor lag, Oxford Knee Score (OKS), Knee Society Score (KSS), pre‐ and post‐operative ambulatory status and post‐operative complications. All evaluations were done by an independent physiotherapist.

**Results:**

Patients treated with this technique achieved reproducible and predictable midterm outcomes with no reported failures and minimal donor site morbidity at a mean follow‐up of 6.9 ± 3.6 years. Of the 29 patients, 8 had no extensor lag, and 21 had a mean lag of 4.2 ± 3.2°. This was a significant improvement from a mean preoperative lag of 66.2 ± 36.4° (*p* value < 0.001). Patients with PT ruptures operated within 3 months (early) of the index surgery demonstrated less extensor lag compared with those where intervention was undertaken after 3 months (late) (3.3 ± 2.4° vs. 9.0 ± 2.2°). This graft construct did not deteriorate over time.

**Conclusion:**

QTPBSTA is a viable alternative for reconstruction of the ruptured PT following primary TKA. Midterm follow‐up at 6.9 ± 3.6 years demonstrated good outcomes, and it may be considered as a reliable option in PT disruption following TKA. The biggest strength of this technique has been a significant reduction in extensor lag.

**Level of Evidence:**

Level IV.

**Abbreviations**
ATA: Achilles tendon allograft
EMA: extensor mechanism allograft
KSS: Knee Society Score
OKS: Oxford Knee Score
PJI: periprosthetic joint infection
PT: patellar tendon
QT: quadriceps tendon
QTPBSTA: quadriceps tendon patellar bone block with semitendinosus augmentation
ROM: range of motion
SAPLT: sitting active and prone passive lag test
SLR: straight leg raises
SM: synthetic mesh
ST: semitendinosus
TKA: total knee arthroplasty

## INTRODUCTION

Patellar tendon (PT) rupture after primary total knee arthroplasty (TKA) is an uncommon but extremely disabling complication with a reported incidence of 0.17–1.4% [8, 17]. Loss of extensor mechanism continuity affects mobility and independence. The role of non‐operative management is limited [9]. Surgical reconstruction is the treatment of choice, and available options include direct repair, hamstring augmentation, quadriceps tendon (QT) autograft, gastrocnemius flap, extensor mechanism allograft (EMA) or Achilles tendon allograft (ATA), and synthetic mesh (SM) [1, 18, 19, 24, 28, 34]. Outcomes using these options report consistently high failure and complication rates. Reconstruction with allografts or mesh although used more frequently has variable outcomes, carrying a higher risk of failure and periprosthetic joint infection (PJI) [5, 31].

Lamberti et al. and Rajgopal et al [21, 28] have reported encouraging short‐term results using the QTPBSTA technique in small case series. Load to failure for PT is higher than QT and significantly higher than single‐strand semitendinosus (ST) graft, providing a biomechanical rationale for combining the autografts [26, 33].

Given the unpredictable outcomes with available techniques, management of PT rupture after primary TKA remains a clinical challenge. This study was undertaken to assess midterm outcomes of PT reconstruction following primary TKA with unresurfaced patellae using the quadriceps tendon autograft with patellar bone block and semitendinosus augmentation (QTPBSTA) technique at a mean follow‐up of 6.9 ± 3.6 years. This is a follow‐up study on our publication in the *Journal of Knee Surgery*, which described this technique and its outcomes at a mean follow‐up of 4 years [28]. The authors hypothesized that the outcomes of their previously described technique using the QTPBSTA option are sustained over time.

## MATERIALS AND METHODS

We retrospectively assessed cases of PT rupture following primary TKA with unresurfaced patellae, managed at a single tertiary care centre between January 2010 and December 2023, after due IRB approval (1850/2025). During this period, a total of 57 post‐TKA patients with complaints of inability to extend their knees, following an episode of trauma, were reviewed.

Inclusion criteria consisted of patients with a confirmed diagnosis of isolated PT disruption following primary TKA with an unresurfaced patella, who were surgically managed with the QTPBSTA technique. This study cohort did not have any mal‐aligned or mal‐rotated knees.

Patients with quadriceps rupture, patellar fractures, associated peri‐prosthetic fracture, PJI and incomplete data were excluded.

From this cohort of 57 patients, those with QT ruptures (*n* = 5) or with patellar fractures (*n* = 16) were excluded from the study. Of the remaining 36 patients with isolated PT ruptures, 3 underwent a direct repair with ST augmentation and were also excluded from the study.

From a cohort of 33 patients who underwent the QTPBSTA procedure, 2 patients passed from unrelated causes (cerebral bleed and cardiac event, 15 and 21 months post‐surgery), and 2 others were lost to follow‐up, 18 and 24 months post‐surgery, leaving us with a final cohort of 29 patients.

**Figure 1:**
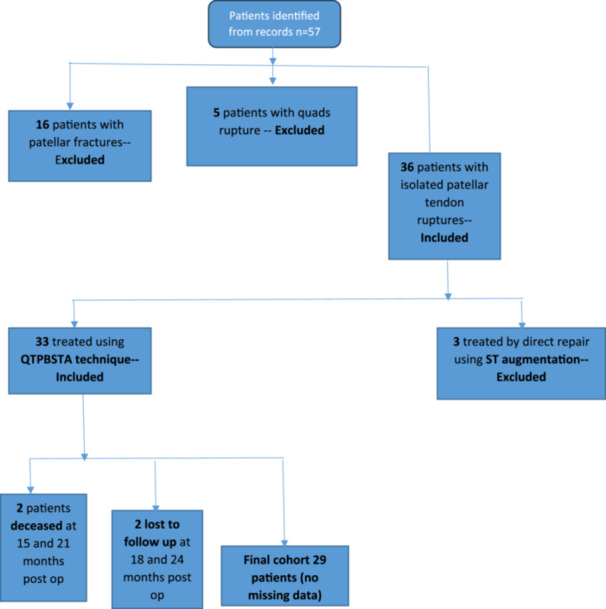
Strobe flow chart.

This is represented in Figure 1, the strobe flow chart.

All the patients were operated on by the senior author. The diagnosis of PT rupture was confirmed based on clinical examination, x‐rays and either ultrasound (US) (*n* = 21) or magnetic resonance imaging (MRI) (*n* = 8) assessments at the time of presentation. The Insall–Salvati ratio was calculated from the lateral radiographs both preoperatively and 6 months post‐operatively [35]. US examination of the knee extensor mechanism was performed using a high‐frequency linear transducer (10–17 MHz) with the patient supine and the knee in 20–30° of flexion. A standardized proximal‐to‐distal approach was used to evaluate the QT, patella, PT and tibial tuberosity in longitudinal and transverse planes. Tendon continuity, echotexture and presence of partial or complete tears were assessed. The suprapatellar pouch was examined for effusion.

**Figure 2:**
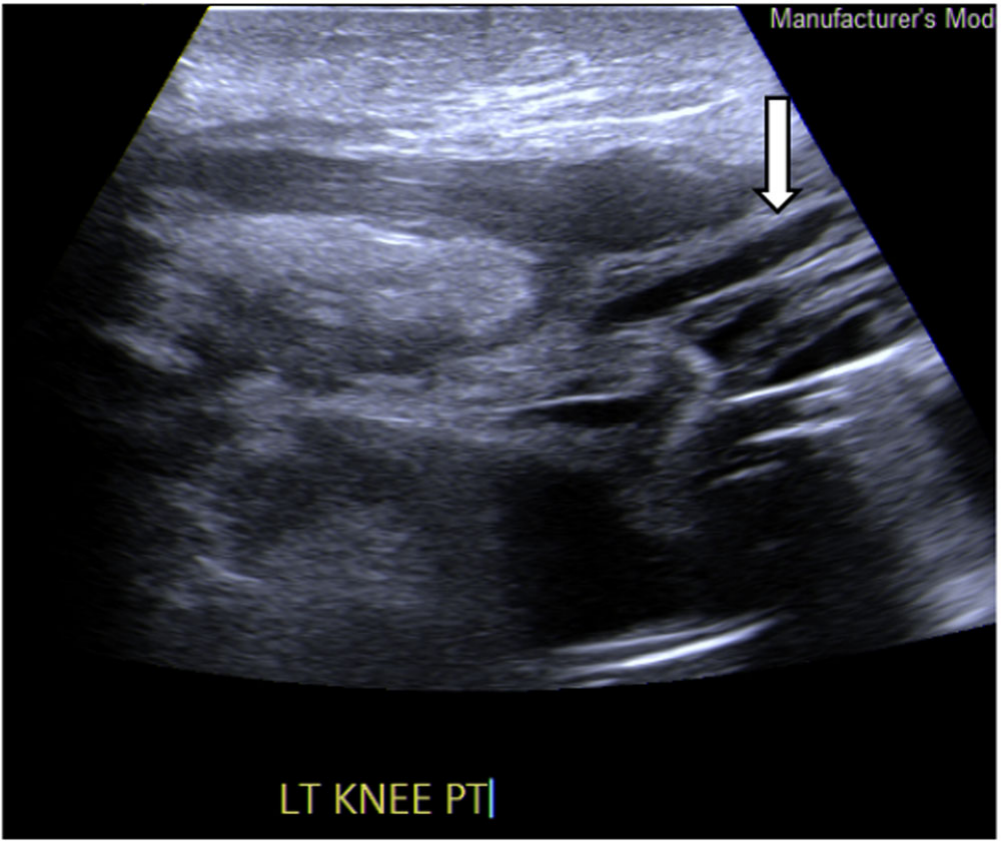
Long‐axis ultrasound image demonstrating ruptured patellar tendon (PT) from its attachment over the tibial tuberosity with an organized haematoma (marked by the arrow) at the retracted tendon site anterior to the tibial tuberosity in the deep infrapatellar bursal region.

Figure 2 demonstrates the ruptured PT preoperatively on a long‐axis US image.

The final cohort of 29 patients included 24 patients presenting early (<3 months from the index surgery) and 5 presenting late (>3 months). In this final cohort of 29 patients, 12 patients underwent our own primary surgeries, and 17 patients were referred from other centres. There was no missing data for any of the study variables; no imputation was performed, and all enroled patients were included in the final analysis.

Of the 29 knees, 16 had cruciate retaining (CR) and 13 had posterior stabilized (PS) implants. Patients who were assessed preoperatively using MRI imaging were checked using the Berger protocol for mal‐alignment, while the remaining were checked intra‐operatively to ascertain correct rotational alignment.

Data collection included pre‐ and post‐operative measurement of extensor lag, Oxford Knee Score (OKS), Knee Society Score (KSS), pre‐ and post‐operative ambulatory status and post‐operative complications.

Extensor lag measurements were done using the sitting active and prone passive lag test (SAPLT) [15] technique using a hand‐held goniometer. Muscle strength was assessed manually using the Manual Muscle testing technique [20].

Viability of the graft was assessed at 24 weeks post‐operatively and at final follow‐up using the same radiological tool as used preoperatively (US = 21 and MRI = 8). US or MRI assessments were done at the 24‐week follow‐up and subsequently at the time of final follow‐ up to confirm viability of the graft.

Failure was defined as an extensor lag of >30° [10] or revision surgery for reconstruction.

### Surgical technique

The QT was exposed using the prior surgical incision. A strip of the tendon and bone block measuring 10 cm in length and 20 mm wide was marked out. The fibres of the vastus medialis and lateralis were teased out to expose the tendon. A full‐thickness QT graft measuring 7.5 cm in length was identified along with a triangular bone block 2.5 cm in length and 15 mm in width and depth, which was harvested taking care to prevent patellar fractures, ensuring at least 6–7 mm residual patellar bone in the trough. A corresponding trough of the same dimensions was made in the region of the tibial tubercle, and this harvested bone from the tibial tubercle was used to graft the patellar defect. The patellar bone block was fixed to the tibial tubercle in a press‐fit fashion and stabilized using two 3.5 mm fully threaded cancellous screws.

The QT was split, and lead sutures were passed through a tunnel in the patella and sutured under maximal tension in full extension to itself. The defect between the two strands was closed using non‐absorbable 2 Ethibond sutures. A distally attached ST graft was harvested and used to reinforce the QT graft and sutured to the medial and lateral retinaculum and remnants of the original PT creating a robust PT graft construct. Graft tensioning in full extension and immobilization for 8 weeks was critical to ensure adequate healing.

**Figure 3:**
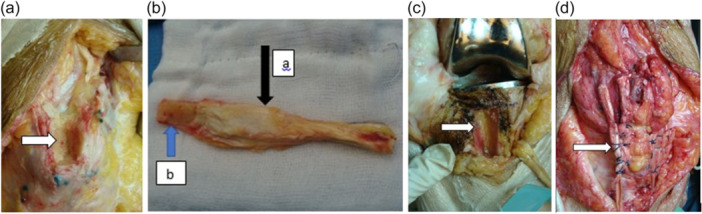
(a–d) Intraoperative pictures. (a) Arrow shows the trough in the region of the tibial tubercle. (b) The arrows show (a) the quadriceps tendon graft and (b) the patellar bone block. (c) Coronal view of the graft trough in the tibial tubercle. (d) Graft in situ with semitendinosus augmentation.

**Figure 4:**
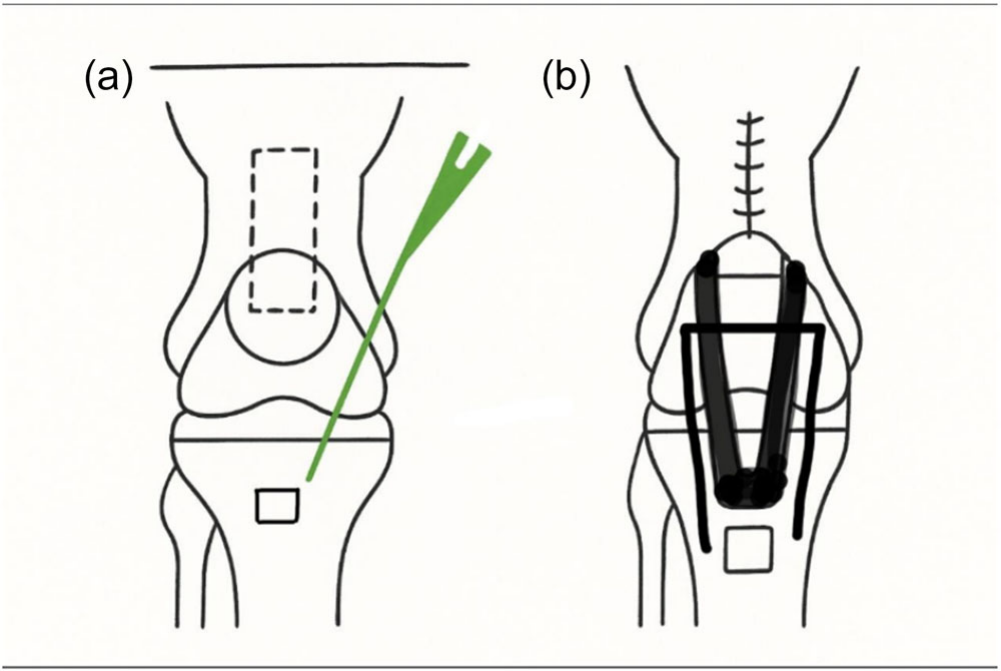
(a and b) Schematic representation of the surgical procedure.

Figure 3a–d is intraoperative pictures demonstrating the surgical procedure, and Figure 4a,b is a diagrammatic representation of the same.

### Post‐operative protocol

Post‐operatively, all the patients were kept non‐weight bearing with the knee immobilized in a long leg brace for 8 weeks. Isometric quadriceps contractions were encouraged, but active straight leg raises (SLR) were not allowed. Partial weight bearing with the leg in a straight knee brace was started at 8 weeks post‐operatively. The straight knee brace was changed to a dial‐a‐lock knee brace at this stage, and gradual range of motion (ROM) exercises were started (0–45°). To begin with, increasing by 15° every week to achieve 90° of flexion by 12 weeks from the date of surgery. Full weight bearing with a stick was started at 12 weeks. Patients were followed up at 3, 8, 12, 16, 24 weeks and annually thereafter. ROM, KSS and OKS were assessed at each follow‐up visit by an independent physiotherapist.

### Statistical analysis

Statistical analysis was performed using IBM SPSS Statistics software (version 23.0; IBM Bangalore). Continuous variables were assessed for normality using the Shapiro–Wilk test. Normally distributed continuous data are presented as mean ± standard deviation (SD), while categorical variables are expressed as frequencies and percentages.

Pre‐operative and final follow‐up continuous outcomes, including extensor lag, OKS and KSS, were compared using the paired Student's *t* test. For comparisons between patients presenting with early (<3 months) versus late (>3 months) PT ruptures, the independent samples *t* test was used for continuous variables. Categorical variables, including ambulatory status and complication rates, were analyzed using the chi‐square test or Fisher's exact test, as appropriate.

Mean differences between pre‐operative and post‐operative outcomes were reported along with their 95% confidence intervals (CIs). A two‐tailed *p* value < 0.05 was considered statistically significant.

Failure was defined a priori as an extensor lag greater than 30° or the need for revision of the extensor mechanism reconstruction. No adjustment for multiple comparisons was performed, given the exploratory nature of this retrospective study. No formal size or power calculation was performed because of the relatively uncommon nature of the injury and the small number of patients in the study cohort.

## RESULTS

The study cohort evaluated 29 patients (20 females and 9 males) of PT rupture following primary TKA, treated by our technique at a mean follow‐up of 6.9±3.6 years. The mean age was 65.9 (±6.1) years. The mean Charlson comorbidity index was 4.33 (±1.02) with a body mass index of 31.4 ± 4.2 kg/m^2^.

The Insall–Salvati ratio improved from a mean value of 2.13 ± 0.34 preoperatively to a mean of 1.09 ± 0.15 with a *p* value < 0.001 at 6 months post‐operatively. The mean preoperative lag was 66.2 ± 36.4°, and at final follow‐up was 4.2 ± 3.2° (*p* value < 0.001) (95% CI). This reduction in extensor lag enabled independent ambulation in 20 of the 29 patients (68.9%). The other nine patients (31.0%) were walking with a stick.

Extensor lag in patients with early ruptures was less than that of those who reported with late ruptures (3.3 ± 2.4° vs. 9.0 ± 2.2°) (*p* value < 0.001).

The preoperative OKS and KSS were 12.4 ± 2.9 and 20.14 ± 4.6, respectively. The OKS and KSS at final follow‐up were 40.7 ± 9.8 and 89.1 ± 3.4 (*p* value < 0.001) (95% CI), respectively. These outcomes are tabulated in Table 1.

**Table 1:** Comparison of outcomes and extensor lag preoperatively and at final follow‐up.

	Pre‐op (mean ± SD)	Final follow‐up (mean ± SD)	Mean ± SD of difference	95% CI of the difference		*p*
				Lower	Upper	
Extensor Lag	66.2 ± 36.4°	4.2 ± 3.2°	61.9 ± 26.4°	51.9	72.0	<0.001*
OKS	12.4 ± 2.9	40.7 ± 9.8	−28.3 ± 9.4	−31.9	−24.7	<0.001*
KSS	20.0 ± 4.5	89.1 ± 3.4	−69.1 ± 4.8	−70.9	−67.2	<0.001*

Abbreviations: CI, confidence interval; KSS, Knee Society Score; OKS, Oxford Knee Score; SD, standard deviation.

* *p* value < 0.05, statistically significant.

QTPBSTA technique has shown reliable outcomes in PT injuries following primary TKA with no failures at a mean follow‐up of 6.9±3.6 years, and significant improvement in PROMs and extensor lag. All patients reported improved ambulatory status with no clinically reported graft site morbidity or implant failures.

**Figure 5:**
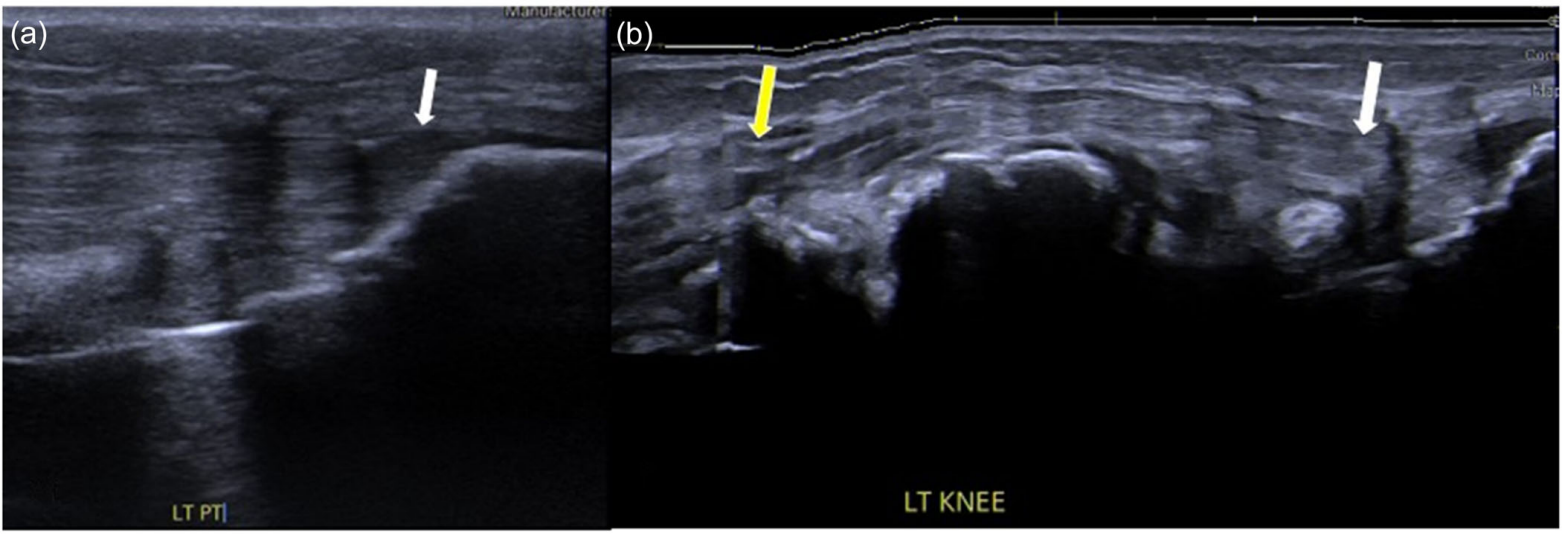
Panoramic ultrasound performed 6 months after surgery demonstrating intact graft with ligamentization extending from (a) anteromedial aspect of quadriceps tendon in the distal portion of the thigh, extending over the patellar tendon and (b) reaching distally up to the tibial tuberosity.

Post‐operative US evaluation done at 6 months post‐operatively demonstrated ligamentization of the graft. None of the cases demonstrated thinning, echogenicity or discontinuity at subsequent follow‐ups. This is demonstrated in Figure 5.

**Figure 6:**
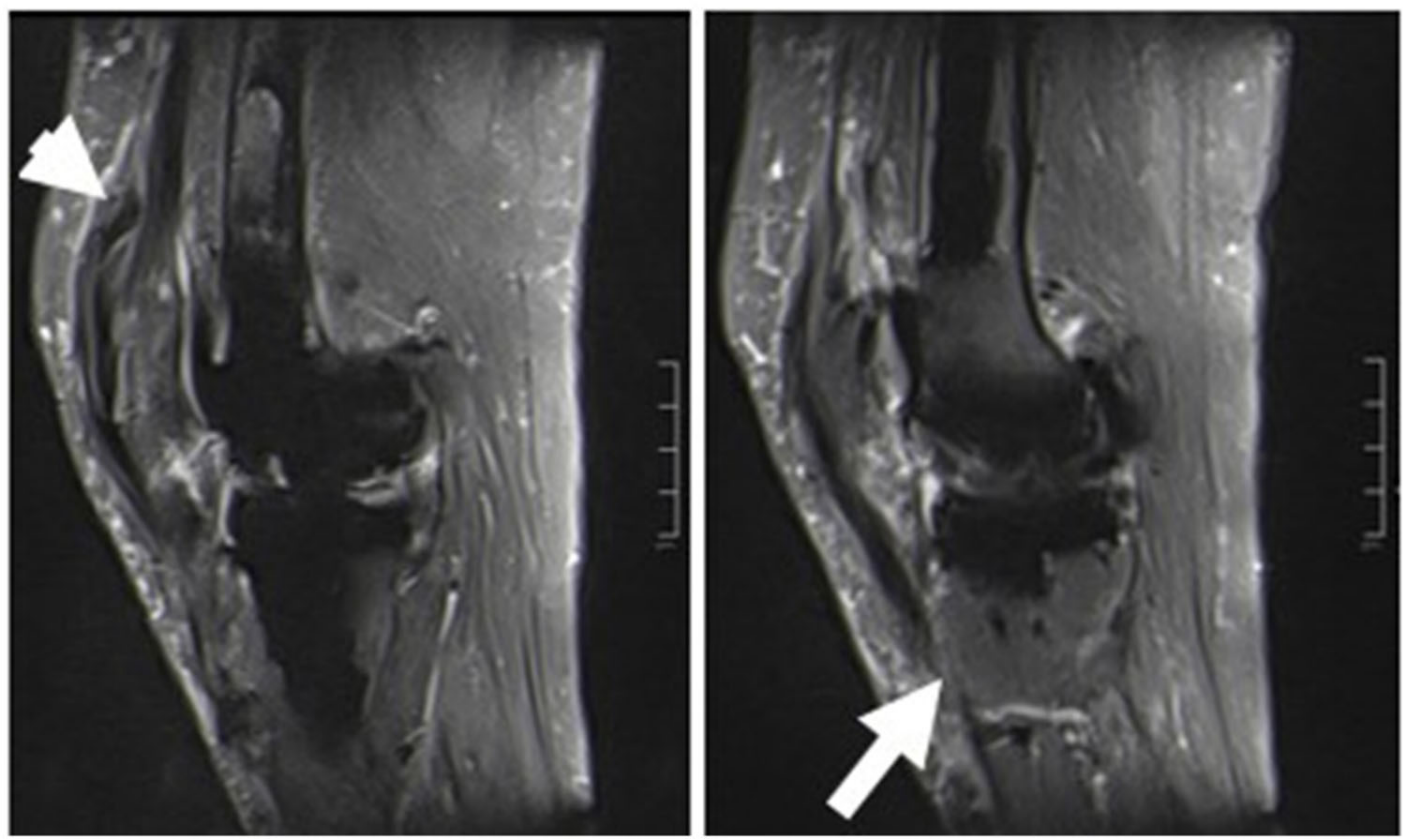
Long‐axis MRI scan performed 7 years after surgery. The graft extending from anteromedial aspect of quadriceps tendon in the distal portion of the thigh extending over the patellar tendon and reaching distally up to the tibial tuberosity is marked by arrows. MRI, magnetic resonance imaging.

**Figure 7:**
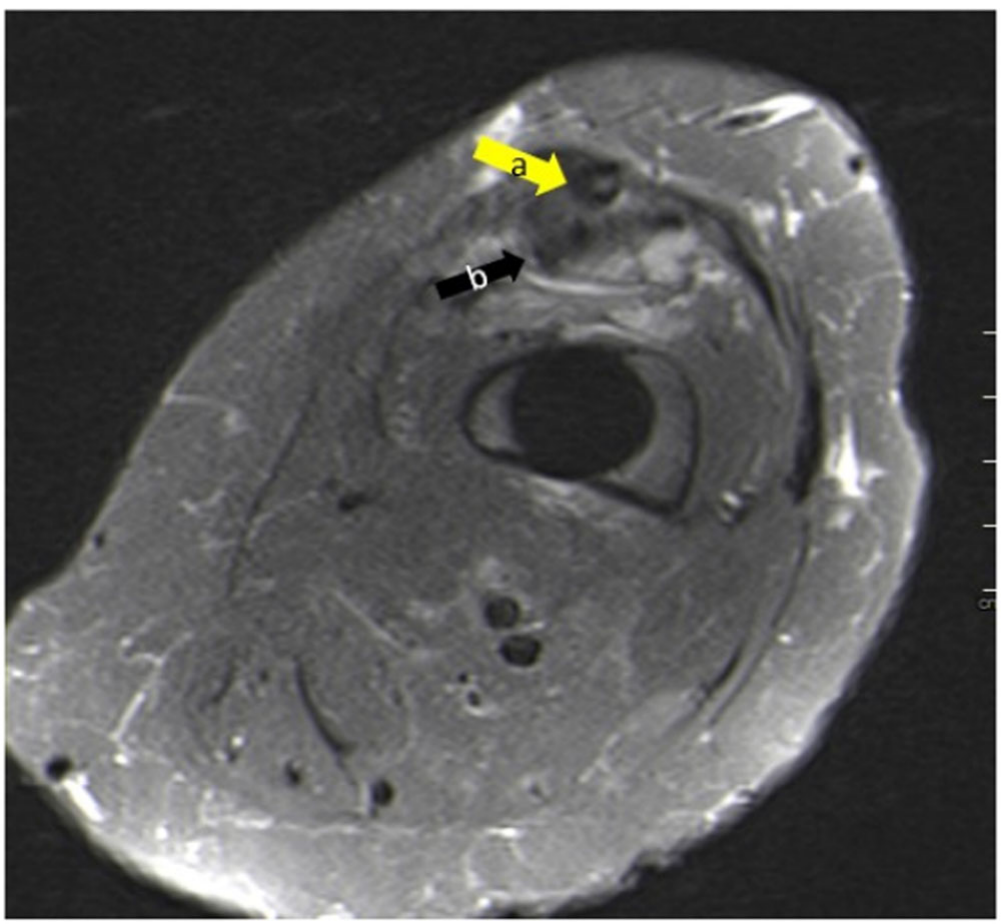
Axial MRI image showing the (a) graft and (b) the intact fibres of the quadriceps. MRI, magnetic resonance imaging.

Long‐axis MRI scan performed at final follow‐up demonstrated an intact reconstructed PT with the graft extending from the patella to its insertion at the tibial tuberosity (Sagittal view Figure 6 and Axial view Figure 7).

One patient developed a patellar subluxation, and one patient developed a PJI associated with delayed wound healing requiring revision TKA. These patients, however, did not require revision of the PT reconstruction.

## DISCUSSION

PT rupture following primary TKA is an uncommon but very disabling condition. The results using the QTPBSTA technique for treatment of this condition demonstrated encouraging outcomes with significant improvements in extensor lag, OKS and KSS, and no clinical failures at a mean follow‐up of 6.9 ± 3.6 years. The rationale for this technique lies in harvesting a QT graft with a patellar bone block, which is fixed press‐fit into the tibial tubercle to promote bone‐to‐bone healing. The construct is further reinforced with the distally attached ST, which augments the repair and adds stability [21, 28].

Chen et al. and Ecker et al. reported on reconstructing the PT in native knees, using a distally attached hamstring tendon. This technique offers support to the PT from both sides, giving it better fixation and possibly better healing potential [13, 16]. Our findings suggest that applying this combined approach in the arthroplasty setting may offer reliable fixation and functional restoration.

PT ruptures occurring within three months of the primary TKA are considered early injuries, and all others are classified as late injuries [14, 34]. Vajapey et al. [34] reported higher complication rates in the early rupture cohort of PT rupture and intervention. This is in contrast to our findings, where we found better outcomes and lower complication rates in the early rupture cohort.

Direct repair of acute ruptures is associated with high complication rates. with Courtney et al. and Li et al. reporting failure rates of 60% and 20%, respectively, using this technique [14, 23]. While articles reporting on allograft usage describe satisfaction rates as high as 89%, they also report failure rates of up to 40% [9, 11, 22]. Long‐term survivorship of EMAs has been reported as only 56.2% at 10 years [29]. SM reconstructions have also yielded inconsistent results, ranging from an 85% survivorship at 2 years [6] to a 57% survivorship at 5 years [4] with a failure rate exceeding 30% [29]. Collectively, these data highlight the variability of outcomes with allografts and mesh.

Failure rates of allograft and SM in the literature are tabulated in Table 2.

**Table 2:** Failure rates of allograft and synthetic mesh in the literature.

Sl. No.	Author	Year	Sample size	Modality	Follow‐up years/months	Failures
1.	Courtney et al. [14]	2018	68	Allograft	24 months	44%
2.	Leopold et al. [22]	1999	7	Allograft		100%
3.	Brown et al. [10]	2015	50	Allograft	57.6 months	38%
4.	Weintraub et al. [36]	2023	10 (Revision EMA)	Allograft	3.65	70%
5.	Baker et al. [6]	2024	123 (all anatomical regions)	Synthetic mesh vs. Allograft	5.3 (±3.2)	57% vs. 52%
6.	Richardson et al. [30]	2024	50	Synthetic mesh vs. Allograft	3.6	26.7% vs. 35%
7.	Anderson et al. [4]	2023	56	Synthetic mesh vs. allograft	3.2	48.2% vs. 53.4%

Abbreviation: EMA, extensor mechanism allograft.

In this series, there were no clinical failures of the QTPBSTA technique of PT reconstruction after primary TKA at midterm follow‐up. This compares favourably with other currently available options. Cadambi et al. reported satisfactory outcomes using hamstring autograft in a series of seven patients [12], and Spoliti et al. observed no failures at 4 years in nine patients reconstructed with ipsilateral hamstring autograft, though nearly half required walking aids [32].

Patients in the present study cohort demonstrated a mean extensor lag of 4.2 ± 3.2° (*p* value 0.001) at midterm follow‐up, representing a clinically meaningful improvement. Extensor lag in patients with early ruptures was less than that of those who reported with late ruptures (3.3° ± 2.4° vs 9.0° ± 2.2°) (p value < 0.001), demonstrating better outcomes in patients presenting with early ruptures.

Outcomes using this technique were favourable relative to other published series, though a direct comparison was not possible. Browne et al. noted persistent extensor lag greater than 10° in patients reconstructed with mesh [9], and Abdel et al. observed functional improvements after mesh reconstruction but did not demonstrate complete restoration of extension [2]. Allograft reconstructions have generally reported higher rates of residual lag, often exceeding 10° [11, 22, 29].

Our data suggests that QTPBSTA restores active extension predictably compared to traditional allograft or mesh methods. Adequate immobilization in extension is necessary to prevent graft elongation and extensor lag [30, 31]. It may serve as an effective alternative option to other reconstructive methods.

Extensor lag using different options (autograft, mesh and allograft) in the literature is tabulated in Table 3.

**Table 3:** Extensor lag using different options (autograft, mesh and allograft) in the literature.

Sl. no.	Author	Year	Sample size	Modality	Follow‐up period (years)	Mean final extensor lag (degrees)
1	Rajgopal et al. [28]	2015	7	QSA	4	5
2	Spoliti et al. [32]	2016	9	Hamstring	3.9	5
3	Hasegawa et al. [18]	2021	4	Polyethylene cable	3.5	0
4	Akgun et al. [3]	2024	14	Hamstring	3.8	5 (±5.3)
5	Abdel et al. [2]	2019	77	Synthetic mesh	4	9 (±14)
6	Masauros et al. [25]	2023	4	Hamstring with partial quadriceps augmentation	1	3.3
7	Baldini et al. [7]	2023	22	Allograft	4	4.6 (±10)

US or MRI scans at 6 months and at final follow‐up showed no evidence of thinning, waviness or structural deterioration, supporting the evidence of the integrity of the repair. This contrasts with the delayed elongation and weakening often observed with allografts, where graft stretching has been linked to persistent lag and functional decline [9, 27]. SM, although not subject to biologic remodelling, has also demonstrated symptomatic lengthening and failure within a few years of implantation [4, 5]. Our imaging findings provide reassuring evidence of early durability, although longer‐term follow‐up beyond one decade will be necessary to confirm whether this benefit is sustained in the long term.

Our findings suggest that this is a promising technique for PT rupture following primary TKA with unresurfaced patella as compared to other available options demonstrating reliable midterm outcomes.

## LIMITATIONS

This study has several limitations. Its retrospective design introduces inherent risks of selection and assessment bias. Although it represents the largest series to date using this technique, the overall sample size remains small with heterogeneous referral sources, limiting statistical power and generalizability. This surgical option is applicable for patients with unresurfaced patellae only. Quadriceps strength was not objectively measured, which is a relevant consideration since quadriceps harvest has been associated with transient weakness in non‐TKA patients [16].

## CONCLUSION

This study demonstrates good outcomes of reconstruction in PT ruptures following primary TKA using the QTPBSTA technique. There was consistent improvement in outcomes such as extensor lag and independent mobility, with a very low complication rate and negligible donor site morbidity. Final extensor lag in patients presenting with early ruptures was significantly less than that in patients presenting with late ruptures of the PT. These outcomes did not deteriorate over time, and this option may be considered as a viable option with predictable outcomes for dealing with this condition.

## AUTHOR CONTRIBUTIONS

**Ashok Rajgopal**: Conceptualization; supervision; validation; writing—review and editing. **Saksham Tripathi**: Data curation; formal analysis; writing—original draft. **Manish Kumar Singh**: Data analysis; statistical support. **Kalpana Aggarwal**: Data curation; writing—review and editing.

## CONFLICT OF INTEREST STATEMENT

The corresponding author is a co‐editor for *Archives of Orthopaedic and Trauma Surgery*. The corresponding author is a consultant for Microport and Shalby. The remaining authors declare no conflicts of interest.

## ETHICS STATEMENT

Institutional Review Board and Ethics Committee (Medanta Institutional Ethics Committee) approval was obtained prior to initiation of the study. The IRB reference number is 1850/2025.

## Data Availability

The data that support the findings of this study are available on request from the corresponding author.
